# Gene expression changes in human cerebral arteries following hemoglobin exposure: implications for vascular responses in SAH

**DOI:** 10.3389/fphys.2025.1529113

**Published:** 2025-04-03

**Authors:** Chathathayil M. Shafeeque, Arif O. Harmanci, Sithara Thomas, Ari C. Dienel, Devin W. McBride, Kumar T. Peeyush, Spiros L. Blackburn

**Affiliations:** ^1^ The Vivian L. Smith Department of Neurosurgery, University of Texas Health Science Center at Houston, Houston, TX, United States; ^2^ D. Bradley McWilliams School of Biomedical Informatics, University of Texas Health Science Center, Fannin, TX, United States

**Keywords:** brain vessels, cerebral arteries, hemoglobin, subarachnoid hemorrhage, vascular injury, ferroptosis

## Abstract

Subarachnoid hemorrhage (SAH), characterized by the presence of hemoglobin (Hb) in the subarachnoid space, significantly impacts cerebral vessels, leading to various pathological outcomes. The toxicity of cell-free Hb released from erythrocytes and its metabolites after SAH causes vasoconstriction and neuronal damage, and correlates with delayed ischemic neurological deficits (DIND). While animal models have provided substantial and invaluable data in the research of aneurysmal SAH, the specific effects of subarachnoid blood on cerebral arteries remain greatly understudied. Here, we describe the changes in the genetic profile of human cerebral arteries exposed to free Hb for 48 h. We performed an *ex vivo* exposure, followed by mRNA sequencing of the vessels. Compared to controls 54 genes were downregulated, and 53 genes were upregulated in human cerebral arteries after Hb exposure. Enrichment analysis identified the ferroptosis pathway as the most significantly affected. Further lipid peroxidation (LPO) assays and elevated *ACSL4* gene expression support a ferroptosis pathway. Additionally, Hb exposure altered key signaling pathways essential for vascular stability (PI3K-Akt, MAPK), modified G-protein signaling mediated by *RGS1/2*, and suppressed key transcription factors such as *KLF5, NR4A1,* and *FOS*. Our results underscore the critical role of Hb in driving pathological responses in brain vessels. Furthermore, our dataset could be valuable for developing interventions after SAH and may help identify the underlying causes of vascular injury.

## 1 Introduction

Subarachnoid hemorrhage (SAH) occurs when blood is released into the subarachnoid space due to the rupture of a cerebral aneurysm or other vascular malformations. Among the numerous components of blood, hemoglobin (Hb) is a key mediator of cellular injury and inflammation following brain hemorrhage insults (Foley et al., 1994). Hb undergoes a series of biochemical transformations, including oxidation and degradation, leading to the release of free heme and iron (Bulters et al., 2018; Macdonald and Weir, 1991). These reactive byproducts have been implicated in oxidative stress, inflammation, and neuronal damage (Bulters et al., 2018). One study has been conducted in mice to understand the genetic changes of brain microvessels after SAH (Tso et al., 2021), however, no studies describe the specific effects of Hb induced genetic changes in human brain vessels. It is important to study Hb-induced gene expression changes in brain vessels to separate out effects on vessels from changes due to inflammation, ischemia, and other SAH pathophysiological aspects. Knowing Hb-specific gene expression changes in vessels may elucidate the mechanistic pathways leading to vasospasm and endothelial dysfunction.

In this study, we have collected leptomeningeal arteries from human brain samples to investigate the specific effects of Hb exposure on cerebral vessels. We hypothesize that Hb exposure induces distinct genetic alterations in human cerebral arteries, contributing to vascular dysfunction and the progression of delayed ischemic injury following SAH. Our work aims to provide insights into the pathophysiology underlying vascular damage in the context of hemorrhagic brain injury.

## 2 Results

While healthy controls would be ideal, harvesting cortical branches in humans is not clinically feasible. Instead, cerebral arteries were obtained from patients undergoing neurosurgical procedures. Vessels from lobectomies for seizure surgeries and tumor resections provided a viable approach for our study. Neurosurgeons took care to collect vessels from areas distant from the pathological lesions to ensure the samples represented “normal” vascular tissue as closely as possible. The vessels were flushed with sterile PBS to remove blood cell contamination. The collected vessel specimens were divided into three groups: T0 controls, vessels processed immediately after collection; T48 controls, vessels cultured in vascular culture medium for 48 h; and Hb treatment, vessels cultured in medium containing 25 µM Hb for 48 h. We compared the transcriptomic changes in vessels and, given that these vessels primarily consist of endothelial and smooth muscle cells, examined the expression of specific markers, as shown in Supplementary Figure S1).

### 2.1 Transcriptomic changes specific to Hb toxicity in human cerebral arteries

**FIGURE 1:**
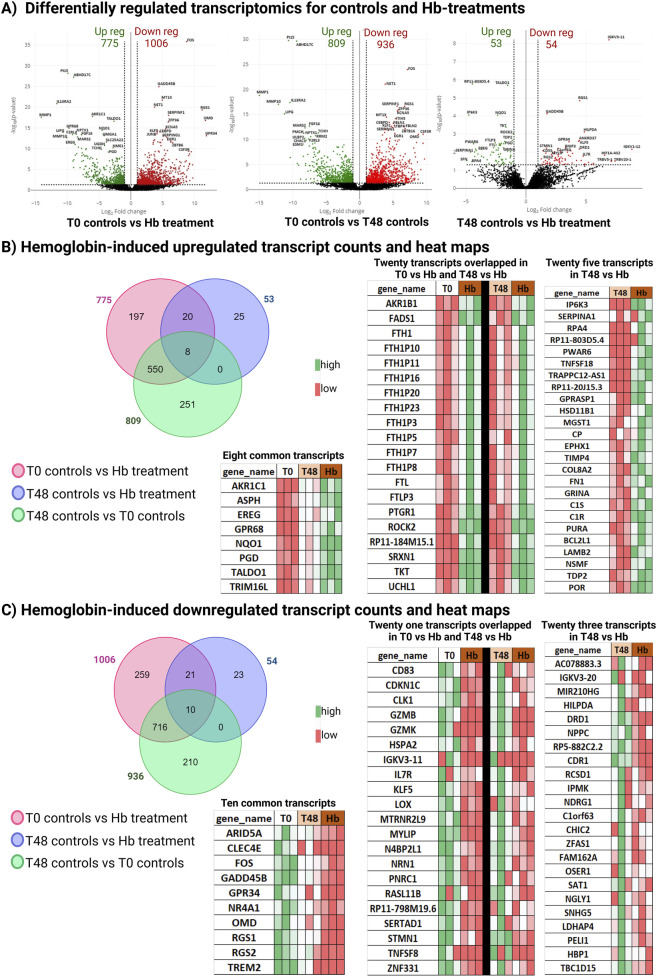
Transcriptomic changes of differentially regulated transcripts. **(A)** Volcano plots for T0 controls (immediately processed) vessels vs. Hb-treated (48 h culture with Hb) vessels, T0 control vs. T48 control, and T48 control vs. Hb treatment; differentially regulated transcripts are represented. **(B)** Venn diagram representing differentially upregulated Hb candidates with heatmap. **(C)** Venn diagram representing differentially downregulated Hb candidates with heatmap.

To identifies the gene cohorts differentially expressed due to Hb toxicity, we compared the Hb-treated vessels (48 h culture with Hb) to both T0 controls (immediately processed) and T48 controls (48 h culture with media). Compared to T0 controls, a total of 1,781 transcripts were differentially regulated, with 775 genes upregulated and 1006 genes downregulated. However, this includes the transcripts changed due to both culture and Hb treatment. So, we compared T0 controls vs. T48 controls, to identify genes that are influenced by the culture media. We observed 809 transcripts upregulated and 936 transcripts downregulated in the human cerebral vessels cultured in media (top ten pathways are shown in Supplementary Figure S2). Upon comparing Hb treatment to T48 controls, we identified 107 differentially regulated transcripts, with 53 upregulated and 54 downregulated in the Hb-injured group (Figure 1A). Out of 53 upregulated genes, 20 were also upregulated in the T0 control vs. Hb comparison, suggesting they increased regardless of culture conditions when treated with Hb. Eight genes overlapped in both the T0 vs. Hb and T0 vs. T48 comparisons, suggesting these genes responded to the initial culture change and were further upregulated by Hb treatment (Figure 1B). Similarly, among the 54 downregulated genes, 21 overlapped with the T0 vs. Hb comparison, and 10 overlapped with both T0 vs. Hb and T0 vs. T48 comparisons (Figure 1C).

### 2.2 Characterization of Hb toxicity-induced genetic changes reveals alterations in apoptosis, oxidative stress, and cell signaling pathways

**FIGURE 2:**
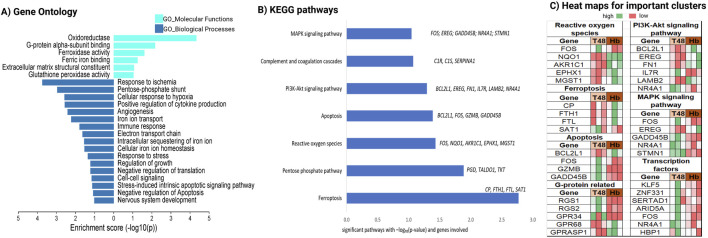
Characterization of Hb toxicity-induced genetic changes via gene enrichment analysis. Enrichment analysis for Hb specific transcripts **(A)** Gene ontology with MF and BP, **(B)** Pathway enrichment with KEGG and, **(C)** heatmap for important cluster of genes.

Next, we performed functional enrichment analysis on the Hb-induced transcriptome from our dataset to identify the cellular functions altered in human cerebral arteries following Hb exposure. KEGG pathway analysis revealed significant enrichment in the ferroptosis pathway, a form of programmed cell death based on iron metabolism (Figure 2B). In animal brain vessels *Cp, Fth*, and *Ftl*, play roles in iron metabolism after SAH (Deng et al., 2023). Similarily these human genes *CP, FTH1, and FTL* were significantly upregulated, and *SAT1* was significantly downregulated after Hb exposure*.* Hb also activated the apoptosis pathway in the cerebral arteries, upregulating *BCL2L1*, an anti-apoptotic gene, indicating that apoptotic resilience in cerebral arteries is mediated through this gene.

Additionally, we found that critical cell signaling pathways such as PI3K-Akt and MAPK were altered following Hb exposure (Figure 2B). Among these, *IL7R*, which can directly activate the PI3K-Akt pathway upon binding *IL7* (Swainson et al., 2007), showed significantly higher expression in the Hb-treated group. *FOS*, a key downstream effector of the MAPK pathway (Yokoyama et al., 2013), was significantly downregulated in the Hb-treated group. *NQO1* and *MGST1*, crucial for reactive oxygen species (ROS) regulation, play direct roles in detoxifying ROS and maintaining cellular redox balance (Figure 2C).

### 2.3 Validation of ferroptosis markers in Hb-treated endothelial cells

**FIGURE 3:**
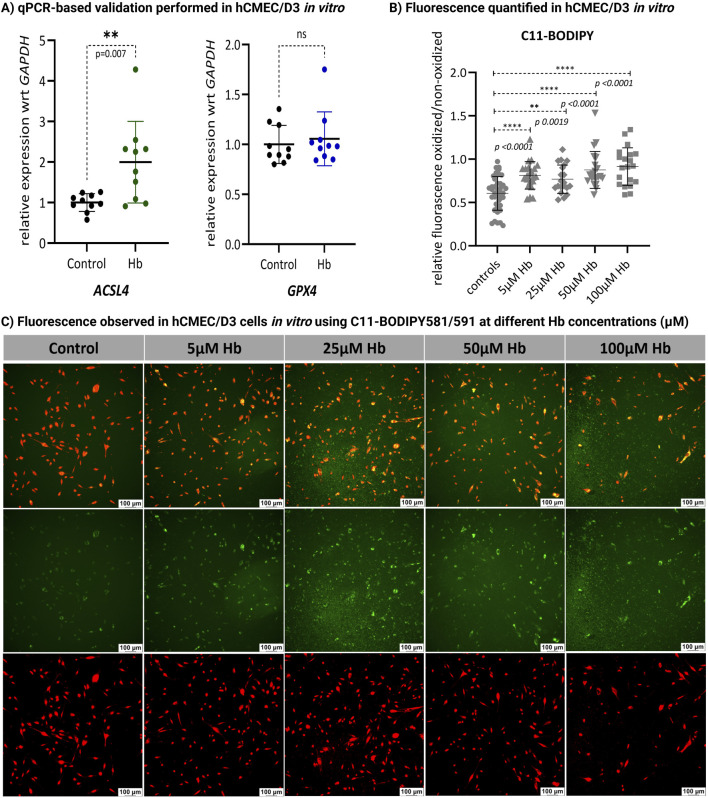
Validation experiments in hCMEC/D3. **(A)** *In vitro* validation using qPCR in human brain ECs (hCMEC/D3) cultured with and without Hb showed significant (t-test) upregulation of *ACSL4* (p = 0.007; n = 10), while *GPX4* expression remained unchanged (ns; n = 10). **(B)** Lipid peroxidation (LPO) in control and Hb-treated brain ECs (hCMEC/D3) was assessed after 24 h of exposure to 5, 25, 50 and 100 µM Hb using C11-BODIPY staining. The relative fluorescence of oxidized (green) vs. non-oxidized (red) signals was quantified (n > 10) using ImageJ software, revealing a significant (t-test) increase in LPO after Hb treatment. (**p = 0.0019; ****p < 0.0001 vs. controls). **(C)** Fluorescence images of control and Hb-treated cells at varying concentrations were captured at ×10 magnification using a Leica Thunder Imager fluorescence microscope. Oxidation is indicated by green fluorescence (center panel), with co-localized fluorescence shown in the top panel.

Although the KEGG pathway analysis highlighted the involvement of ferroptosis related genes (e.g., *CP, FTH1, FTL*), the key ferroptosis markers *ACSL4* and *GPX4* did not show any significant changes in the human vessel RNA-seq data. This discrepancy may be due to culture-induced changes in the expression of these genes, as shown in Supplementary Figure S5. In human vessels, endothelial cells (ECs) provide a relevant model for studying the role of ferroptosis in various pathophysiological contexts, particularly its effect on endothelial dysfunction (Yuan et al., 2022). To further validate these findings, we examined the expression of these genes in human cerebral microvascular endothelial cell lines, *hCMEC/D3* using qPCR. The cells were cultured *in vitro* and treated with 25 µM Hb. We observed a significant (p = 0.007) upregulation of *ACSL4* expression after Hb exposure in ECs, while *GPX4* did not show any significant difference (Figure 3A). These results partially support our hypothesis that Hb exposure may trigger ferroptosis in brain ECs. To further validate ferroptosis, we performed C11-BODIPY staining (Pap et al., 1999) in brain ECs to evaluate lipid peroxidation (LPO). By quantifying the ratio of oxidized to non-oxidized cells, we observed a significant increase in LPO in the brain ECs after 24 h of Hb treatment compared to the control. Hb concentration from 5µM–100 µM showed significant increase in LPO (Figure 3B).

## 3 Discussion

Previous studies suggest that Hb can trigger cytotoxicity and injury in various brain cell types (Wang et al., 2002; Imai et al., 2019; Sadrzadeh et al., 1987) and in ECs (Meguro et al., 2001a, 2001b). Ferroptosis, characterized by disruptions in iron metabolism and oxidative stress, plays a 161 crucial role in the pathological processes underlying early brain injury following SAH (Ge et al., 2024; Kang et al., 2024). Additionally, study conducted by Imai et al. (2019) have reported ROS over production and iron induced cell death in brain ECs following exposure to 25 μM Hb. However, the genetic changes caused by Hb exposure are unknown. Understanding how exposure to Hb alters gene expression in human cerebral arteries is crucial, as it may shed light on the link between SAH-induced vascular injuries and the risk of developing poor outcomes.

Our transcriptomic data provides compelling insights. By performing the assay on both freshly collected and 48 h cultured vessels, we subtracted the confounding effect of culture conditions on gene expression. This approach allowed us to isolate the distinct genetic alterations caused by Hb in human cerebral arteries, independent of cumulative effects from the immune response or ischemia. This focus on Hb-specific changes is crucial for developing targeted therapies. As expected, the analysis revealed changes in genes associated with iron regulation, iron binding, oxidation state modulation, and ferroptosis processes (Figure 2A). *ACSL4* is a key regulator of ferroptosis through phospholipid remodeling (Doll et al., 2017), and we observed an increased expression of *ACSL4* in human brain ECs. Additionally, our findings indicate that Hb induces LPO (Figures 3B, C), further supporting the activation of ferroptosis in vessels exposed to Hb. Given its role as a key driver of cell death in this context, further research is needed to elucidate its regulatory mechanisms (Deng et al., 2023).

Exposure to Hb affected several key signaling pathways, notably PI3K-Akt and MAPK, which govern crucial cellular functions such as survival, angiogenesis (Karar and Maity, 2011), barrier integrity (Kilic et al., 2006), and inflammation—all of which are essential for maintaining brain vascular health and addressing conditions like SAH. Specifically, the MAPK pathway is critical for managing cellular survival and stress responses and is integral to controlling pro-inflammatory cytokine production and mediating the inflammatory response (Zhang and Liu, 2002). In our study, we observed upregulated complement cascade events following Hb exposure (Figures 1B, 2B). MAPK activation has been linked to cerebrovascular receptor upregulation, contributing to increased vasoconstriction in human cerebral arteries (Ansar et al., 2013). Furthermore, MAPK inhibition has been shown to alleviate SAH-induced vascular dysfunction in animal models (Edvinsson and Krause, 2024) highlighting its potential to mitigate SAH-mediated vascular dysfunction.

The PI3K/Akt pathway is central to regulating cell survival (Glaviano et al., 2023), growth, and metabolism (Xie et al., 2019) and is also known for its role in stress responses. In the SAH-injured brain, PI3K/Akt activation has been associated with neuroprotection, suggesting that it may enhance neuronal survival by upregulating *PI3K, p-AKT,* and *BCL2* expression (Dienel et al., 2021). We observed an increased expression of the anti-apoptotic gene *BCL2L1*, a downstream component of the PI3K/Akt pathway (Figure 2C), in the Hb-exposed group, suggesting a defense mechanism aimed at enhancing cell survival under Hb stress. Concurrently, Hb-induced reduction of MAPK pathway genes alongside elevated ROS levels (Figure 2C) indicates an adaptation for cell survival, with reduced apoptosis supporting resilience under stress.

Our data suggest a complex interplay among ferroptosis, the PI3K/Akt pathway, and MAPK signaling, forming a regulatory network that is influenced by Hb exposure in human brain vessels. The ferroptosis-PI3K/Akt axis is widely recognized as a critical pathway in modulating cell death mechanisms. Recent studies have targeted this axis in mouse models of SAH to attenuate early brain injury and reduce neuronal damage. Additionally, the PI3K/Akt pathway has been extensively studied for its crucial role in mitigating secondary brain injury by reducing BBB permeability, brain edema, and neurological dysfunction (Liu et al., 2024; Zheng et al., 2025). Equally, the MAPK pathway is integral to ferroptosis, with studies indicating that lipid ROS can activate the p38 MAPK pathway, thereby promoting ferroptotic cell death. Previous research has also underscored the importance of the MAPK pathway in SAH, suggesting that early inhibition of vascular MAPK signaling can prevent delayed cerebral ischemia (Kang et al., 2024; Zille et al., 2017). While prior studies have primarily utilized mouse models or cell lines, our investigation using human vessels highlights the significance of this axis in human brain vessels. Further exploration of these mechanisms could aid in identifying therapeutic targets to counteract ferroptotic damage, enhance cellular resilience, and promote vascular repair following SAH.

Additional relevant findings included changes in transcription factors and G-protein signaling genes. We observed a downregulation of key transcription factors, including *KLF5, FOS, NR4A1, HBP1, ARID5A, SERTAD1,* and *ZNF331*, all of which are crucial for regulating cell proliferation, differentiation, survival, stress responses, inflammation and metabolic changes. Disruptions in G-protein signaling-related genes have been linked to endothelial dysfunction (Osei-Owusu et al., 2012) and cognitive impairments in Alzheimer’s pathologies (Hadar et al., 2016). Notably, Hb exposure resulted in decreased levels of *RGS1* and *RGS2*, which may also be associated with cognitive complications related to SAH.

This study reveals a key mechanism of vascular injury post-hemorrhage, providing novel genetic insights in human cerebral arteries. Our findings underscore the therapeutic potential of targeting the ferroptosis, MAPK and PI3K/AKT pathways to reduce Hb-induced endothelial damage. Further exploration of short- (6 h, 24 h) and long-term (>48 h) gene expression changes, particularly in lncRNAs and pseudogenes (Supplementary Figure S3), could offer valuable insights. The overlap between Hb-treated vessel data and mouse SAH data (Tso et al., 2021) identified three genes (*CP, Nr4A1, UCHL1*) with consistent expression patterns (Supplementary Figure S4), supporting the relevance of our findings by demonstrating cross-species conservation of molecular responses to Hb toxicity. This consistency in human conditions and experimental models further strengthens the translational value of our results.

## 4 Conclusion

Data analysis identified specific genes that change expression when in contact with Hb, despite patient-to-patient variability. Further enrichment analysis revealed significantly altered pathways, such as MAPK, PI3K-Akt, and ferroptosis pathways. Our results provide a translational reference to better understand the molecular underpinnings of Hb-based injury to cerebral vessels. These findings can significantly impact our understanding of SAH pathophysiology and could pave the way for novel interventions to mitigate the harmful effects of Hb and enhance recovery in hemorrhagic stroke patients.

## 5 Limitation

Obtaining vessels from patients with SAH is not possible as the brain tissue is preserved. Some investigators have looked at aneurysm dome sections from SAH patients, though this tissue would be pathological due to the aneurysmal changes, and would not reflect the typical vascular response to SAH. Our findings, which suggest inflammatory and other signaling events similar to those in stroke, further support the validity of our observations despite the heterogeneity of the source tissue.

## 6 Materials and methods

### 6.1 Sample collection

Fresh leptomeningeal cerebral arteries were collected from patients who underwent elective surgery for brain tumor resection or lobectomy following consent and enrollment in an IRB-approved protocol (HSC-MS-20-0240). Cortical branches over the hemisphere surface (diameter ∼0.5 mm) were dissected and removed by the operating neurosurgeon, transferred to the culture media (DMEM + Vasculife), and taken to the lab and processed immediately. The vessel lumen was flushed with sterile PBS to remove blood cell contamination. Arteries were cut into three pieces of approx. 1.5 cm each and were cultured in vessel maintaining media 50% DMEM (GenDepot) with 50% VascuLife (Lifeline cell technology) plus 5% FBS (GenDepot), plus 1% antibiotic, antimycotic (GenDepot) and 0.1% Normocin (InvivoGen). All selected samples were from patients aged 28–59 years with a diagnosis of epilepsy or tumor (Table 1).

**TABLE 1:** Sample details on the patient with group, surgery details and age of individuals.

Group	Sample ID	Surgery	Gender	Age
T0 control	T0_01	Tumor resection	Male1	40
	T0_02	Glioma	Male2	58
	T0_03	Tumor resection	Male3	58
T48 control	T48_01	Tumor resection	Male4	59
	T48_02	Glioma	Male2	58
	T48_03	Epilepsy	Female1	38
Hb treatment	Hb_01	Tumor resection	Male1	40
	Hb_02	Epilepsy	Female1	38
	Hb_03	Glioma	Male5	28

### 6.2 Human vessel processing and Hb treatment

Dissected arteries were processed as T0 vessel control (vessels processed for RNA extraction within 30 min of resection), T48 vessel control for 48 h of culturing in media, and Hb vessels for 48 h in culture with Hb treatment. Human Hb (64,500 Da; Sigma-Aldrich, St. Louis, Missouri, USA) was prepared in a 1X PBS stock solution and then diluted to 25 µM using culture media. The media was replaced after 24 h with freshly prepared Hb-containing media.

### 6.3 RNA isolation for integrity check

Total RNA isolation was performed using RNeasy® Mini Kit (Qiagen CatNo:74,104), for which the initial sample mincing was carried out on dry ice with sterilized, pre-chilled blade and pipette tips to avoid degradation of RNA, then transferred to RLT lysis buffer containing beta-mercaptoethanol. Other than this initial set of sample processing, the rest of the steps, including DNase-I treatment, were followed as instructed in the protocol. Once the isolation was done, the sample was sent to the sequencing facility for a quality check using Qubit and Bioanalyzer. Samples with recommended quantity and integrity have been used further for cDNA conversion and library preparations using SMART-Seq V4 PLUS (Takara Bio USA).

### 6.4 RNA sequencing and post analysis

RNA sequencing has been performed using the NovaSeq platform using UTHealth Houston core facility, where 150 PE sequences have been carried out. Fastq files generated were further analyzed manually using the RNA Detector platform (La Ferlita et al., 2021). HISAT2 alignment has been executed and mapping reads of all files were ≥79%, then differential expression performed using DESeq2. Comparative analysis between T0, T48 and Hb vessels has been done. Gene with p-value <0.05 found by DESEq2 were categorized as differentially expressed.

### 6.5 Gene and pathway enrichment analysis

**FIGURE 4:**
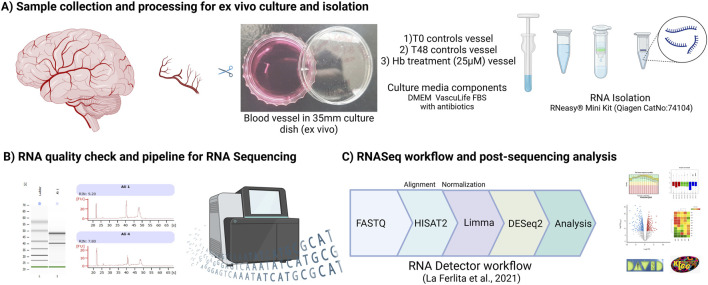
Methodology representing **(A)** Sample collection and processing to RNA isolation, **(B)** Quality check and integrity of RNA assessed using Bioanalyzer **(C)** Sequencing and post data analysis pipeline.

Differentially regulated transcripts were analyzed for gene and pathway enrichment with DAVID (Huang et al., 2009; Sherman et al., 2022), we have used differentially regulated candidates (both up and down) in DAVID to find out gene ontology (GO) and pathway analysis. Detailed ontology terms, molecular functions, biological processes, and cellular components along with KEGG and Reactome Pathway analysis were figured out. GO analysis representation was prepared using MS-Excel (LTSC Professional Plus 2021). Overall methodology is represented here (Figure 4), illustrations and images were Created with BioRender.com (licensed to UT Health Houston).

### 6.6 *In vitro* culture of hCMEC/D3 for ferroptosis validation

We have cultured hCMEC/D3 (CEDARLANE, CELLutions Biosystems Inc.), brain ECs, *in vitro* in a 12 well plate and upon 70% confluency, cells were treated with 25uM Hb prepared stock in 1X PBS and diluted to 25 µM by using DMEM media (GenDepot) with 5% FBS (GenDepot) plus 1% antibiotic, antimycotic (GenDepot) and 0.1% Normocin (InvivoGen). Media was replaced with fresh media after 24 h, and cells were harvested 48 h post-treatment to replicate our culture condition with the human vessel.

We custom-designed primers for *ACSL4* and *GPX4* using Primer3 software, with *GAPDH* serving as the housekeeping gene. The oligonucleotides were synthesized by IDT (Integrated DNA Technologies), and the following sequences were used: *ACSL4* forward primer (TCCAAGTAGACCAACGCCTT) and reverse primer (TATGTGTCCTTCGGTCCCAG); *GPX4* forward primer (GCCAGGGAGTAACGAAGAGA) and reverse primer (CAGCCGTTCTTGTCGATGAG); and *GAPDH* forward primer (CCAGAACATCATCCCTGCCT) and reverse primer (CCTGCTTCACCACCTTCTTG).

To further validate the involvement of ferroptosis, we assessed LPO in hCMEC/D3. Brain ECs were cultured in a 48-well plate, and the next day, they were treated with varying concentrations, i.e., 5, 25, 50 and 100 µM of Hb. After 24 h of Hb treatment, LPO was evaluated using a fluorescence assay with the fluorophore C11-BODIPY 581/591 (Invitrogen). Upon oxidation, this fluorophore results in a changing the fluorescence from red to green. Brain ECs were incubated with 1 µM C11-BODIPY for 30 min at 37°C, followed by washing with PBS. The cells were imaged at ×10 magnification using a Leica Thunder Imager fluorescence microscope. Quantification was performed in ImageJ by measuring the mean fluorescence intensity ratio of oxidized (green) to non-oxidized (red) cells. Statistical significance was assessed using an unpaired t-test in GraphPad Prism (version 10.0.2).

## Data Availability

The mRNA sequencing data generated and analyzed during this study have been submitted to the NCBI Gene Expression Omnibus (GEO) database. These data are accessible through GEO accession number GSE271989.
