# Supplementary figures and images for: Gene expression changes in human cerebral arteries following hemoglobin exposure: implications for vascular responses in SAH
Source: Front Physiol. 2025 Apr 3;16:1529113. doi: 10.3389/fphys.2025.1529113 (PMC12003393; doi:10.3389/fphys.2025.1529113)

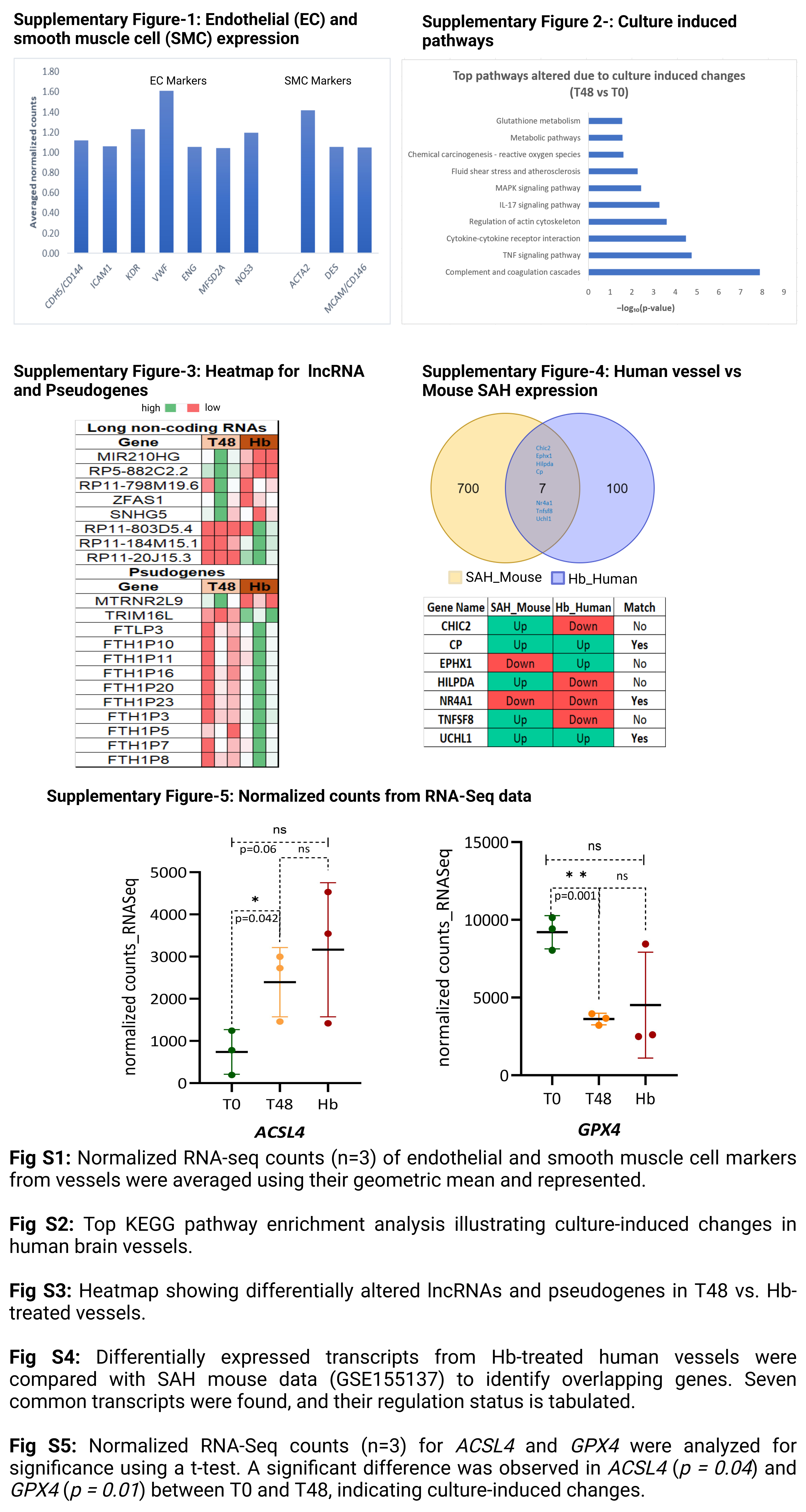

Supplement: Supplementary file 1 [file Image1.jpeg]
